# Provision of School-based Preventive Oral Health Services to Medicaid Beneficiaries

**Published:** 2005-12-15

**Authors:** Anne R Redmond, Nancy Martin

**Affiliations:** Bureau of Health, Department of Health and Human Services, Augusta, Me; At the time this work was completed, Ms Redmond was affiliated with the Public Health Prevention Service, Centers for Disease Control and Prevention, Atlanta, Ga.; Oral Health Program, Department of Health and Human Services, Concord, NH

## To the Editor:

Children from families who qualify for Medicaid, although more likely to receive dental care than uninsured children (1), are less likely to receive dental care than children from middle-income and upper-income families (2). School-based or school-linked oral health programs can play a key role in facilitating regular access to preventive oral health services for children participating in the Medicaid program (3). The New Hampshire Department of Health and Human Services recently assessed the extent to which Medicaid participants are being served through school-based oral health programs that treat children without access to dental services.

### Methods

We analyzed data from all school-based oral health programs in New Hampshire that billed Medicaid for preventive oral health services during July 2000 through June 2003. The number of Medicaid children served by year and service type was examined. All children in selected grades (ranging from kindergarten through grade 12 with grades one through three consistently served) in participating schools were eligible for oral health services. Preventive services were provided only to children who had not seen a dentist in the past year and who had parental consent. Preventive services included prophylaxis, fluoride application, oral health education, and sealants, which were reimbursed at the following intervals: prophylaxis, twice per year; fluoride application, once per year; oral health education, once every 3 years; and sealants, once every 5 years. Because children were eligible for services each year, total counts might have included the same beneficiaries more than once.

### Results

During July 2000 through June 2003, 6 (40.0%) of 15 school-based oral health programs geographically dispersed across the state billed Medicaid for preventive oral health services. There were 25,895 children eligible for oral health services at schools served by the six school-based oral health programs. Of these, 10,859 (41.9%) were screened to determine oral health needs, 2739 (10.6%) received preventive services, and 1024 (4.0%) were Medicaid beneficiaries for whom a bill was submitted (Figure). The majority of the 1024 Medicaid beneficiaries received prophylaxis (75.7%), fluoride application (75.0%), and oral health education (67.2%) (Table); only 4.3% received sealants. Overall, New Hampshire school-based oral health programs billed Medicaid for 1024 (37.4%) of 2739 children who received preventive services from 2000–2003.

**Figure:**
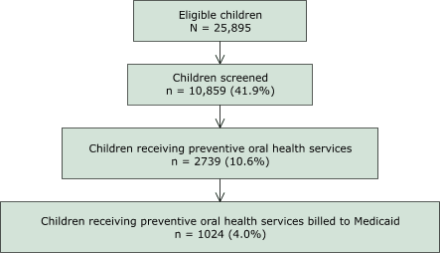
Delivery of school-based oral health services to children enrolled in Medicaid, New Hampshire, July 2000 through June 2003.

### Discussion

A substantial number of Medicaid beneficiaries received preventive oral health services through New Hampshire school-based programs. The majority of Medicaid beneficiaries received prophylaxis, oral health education, and fluoride application. A smaller proportion of beneficiaries received sealants, in part because only three of the six school-based programs included in this study were providing this service. Challenges exist in delivering preventive services, including obtaining parental consent and Medicaid billing information and meeting the state requirement for an examination by a dentist before sealant placement. School nurses often play a role in communicating with parents about school-based oral health services. Some programs have designated staff to work individually with parents to gain consent and Medicaid information. Because sealants are one of the most effective methods of preventing tooth decay (3,4) and school-based sealant programs can reduce oral health disparities among children (5), a 3-year statewide sealant project has been established in collaboration with the New Hampshire Dental Society and volunteer dentists to perform on-site dental examinations and deliver school-based sealant services. Children not eligible for sealants because of untreated decay are linked to area dentists for restorative care. School-based programs provide access to and provision of preventive oral health services for hard-to-reach populations, contributing to increased oral health and the prevention of tooth decay. Reimbursement from Medicaid presents an opportunity for oral health programs to leverage financial support to enhance services.

## Figures and Tables

**Table:** Medicaid Beneficiaries Served Through School-based Oral Health Programs, by Type of Service, New Hampshire, July 2000–June 2003

**School Year**	**No. of Medicaid Beneficiaries**	**Prophylaxis**	**Fluoride Application**	**Oral Health Education**	**Sealants**

		**No. (%)**	**No. (%)**	**No. (%)**	**No. (%)**
2000–2001	280	209 (74.6)	204 (72.9)	205 (73.2)	5 (1.8)
2001–2002	307	258 (84.0)	259 (84.4)	209 (68.1)	11 (3.6)
2002–2003	437	308 (70.5)	305 (69.8)	274 (62.7)	28 (6.4)
**2000–2003**	1024	775 (75.7)	768 (75.0)	688 (67.2)	44 (4.3)
